# Simultaneous Achievement of Low Loss, Large Effective Mode Area and Wide Transmission Band Hollow-Core Anti-Resonant Optical Fibers

**DOI:** 10.3390/s25103003

**Published:** 2025-05-09

**Authors:** Min Liu, Yingqi Cui, Xiangyu Hua, Wenjun Ni, Perry Ping Shum, Lei Huang

**Affiliations:** 1 School of Microelectronics and Communication Engineering, Chongqing University, Chongqing 400044, China; liumin@cqu.edu.cn (M.L.); 202212131060t@stu.cqu.edu.cn (Y.C.); 202212131115@stu.cqu.edu.cn (L.H.); 2 Hubei Key Laboratory of Intelligent Wireless Communications, Hubei Engineering Research Center of Intelligent Internet of Things Technology, College of Electronics and Information Engineering, South-Central Minzu University, Wuhan 430074, China; 2024110198@mail.scuec.edu.cn; 3 Department of Electrical and Electronic Engineering, College of Engineering, Southern University of Science and Technology, Shenzhen 518055, China; shenp@sustech.edu.cn

**Keywords:** hollow-core anti-resonant optical fiber, confinement loss, dispersion, mode field area, transmission bandwidth

## Abstract

A novel nested structure of hollow-core anti-resonant optical fiber is proposed to achieve low loss, large effective mode area, and wide transmission band simultaneously in the near-infrared range of 1200–2200 nm. It is composed of six elliptical cladding tubes nested with six large circular cladding tubes, and six small circular cladding tubes are introduced in the gap of the elliptical tubes. The transmission characteristics of the hollow-core anti-resonant optical fiber are numerically investigated using the full-vector finite element method. The effects of structural parameters such as the cladding tube thickness and the tube diameters on the fiber transmission characteristics are analyzed in detail. The results indicate that within the wavelength range of 1200–2200 nm, the confinement loss remains below 0.017 dB/km, and the minimum confinement loss can be as low as 1.2 × 10^−4^ dB/km at 1500 nm. The effective mode area remains as large as ~1142.5 μm^2^. It should be noted that in the wide wavelength range of 1000 nm, the dispersion exhibits excellent characteristics ranging from 0.7 to 1.4 ps/(nm·km). Our fiber can find potential applications in ultra-long-distance and ultra-high-power transmission systems with a wide operating wavelength band.

## 1. Introduction

Hollow-core anti-resonant fiber (HC-ARF) is a type of hollow-core fiber that offers advantages over other types with the features of simple structure and utilizing air as the material for transmitting light. This leads to excellent characteristics such as low dispersion, low time delay, and high damage threshold [1]. Due to its unique merits, HC-ARFs have become a hot topic in the field of optical fibers and have a wide range of applications, such as fiber sensing, laser transmission [2], mid-infrared lasers [3], etc. With the increasing requirements of optical transmission systems, such as longer transmission distances, faster transmission bit rates, and high-power delivery, it is necessary to dedicate efforts toward exploring high-performance hollow-core anti-resonant fibers.

Currently, great progress has been made on the structure of HC-ARFs to realize better performance, including low confinement loss and high birefringence, etc. In 2018, a nested-tube anti-resonant fiber was proposed, achieving a confinement loss of 2 dB/km at 1512 nm and a loss below 16 dB/km in the wavelength range of 1302–1637 nm [4]. Also, a nested-type HC-ARF consisting of six nested tubes was proposed to achieve a confinement loss of 1.3 dB/km at 1450 nm and a loss below 1.4 dB/km in the wavelength range of 1420–1460 nm [5]. In 2019, Bradley et al. further optimized the structure and fabricated a nested-type fiber with a loss of only 0.65 dB/km in the C and L communication bands [6]. Also, in 2019, a nested HC-ARF composed of six nested tubes was designed, realizing a loss below 10 dB/km in the wavelength range of 1240–1940 nm, with the lowest loss of 6.6 dB/km at 1550 nm [7]. In 2020, a nested HC-ARF was realized, consisting of six nested tubes with an ultralow loss of 0.32 dB/km in the wavelength range of 1530–1625 nm [8]. In 2021, a nested structure with five cladding tubes was fabricated, with the loss reduced to 0.22 dB/km at 1625 nm [9], and a connected-ring HC-ARF was designed with a minimum loss of 3.69 × 10^−3^ dB/km [10]. In 2022, a three-nested structure was proposed to achieve a loss below 0.174 dB/km in the wavelength range of 1530–1565 nm and below 0.22 dB/km in the wavelength range of 1260–1360 nm, respectively [11]. In 2023, a novel crescent-shaped core HC-ARF was introduced to realize a loss of less than 0.1 dB/km over a band range of 600 nm and a significant birefringence of less than 10^−5^ through asymmetric shaping [12]. A new five-tube nested double C-type HC-ARF was realized to obtain single-polarization single-mode transmission at 1550 nm with a bandwidth of 150 nm and an ultra-low confinement loss of 2.8 × 10^−3^ dB/km [2]. A negatively curved core AR-HCF was studied to realize a high birefringence of 1.3 × 10^−4^ and a low confinement loss of 6.1 dB/km at 1550 nm [13]. Still in 2023, a HC-ARF with a nested and double-cladding structure was presented to obtain the minimum confinement loss of 4 × 10^−4^ dB/km at 1550 nm and a high-order mode loss ratio of up to 20,000 [14].

In order to further optimize the performance of HC-ARF, a novel nested HC-ARF is proposed in this paper to realize low confinement loss and a large effective mode area with a wide operating wavelength range simultaneously. In the cladding region, six elliptical tubes are combined and nested with six large circular tubes, and six small circular tubes are introduced in the gaps of the elliptical tubes. Different cladding tube thicknesses and cladding layer thicknesses are utilized to reduce the confinement loss, enhance the effective mode area, and increase the transmission band. The effects of different structural parameters on the confinement loss and the effective mode area are investigated in detail according to the priority of their influences on performance.

## 2. Structure Design and Basic Theory

The structural design is considered in the order of decreasing confinement loss, enlarging the effective mode area, and widening the operating wavelength band. The involved structural parameters include the number of cladding tubes N, the thicknesses of the elliptical and circular cladding tubes, the thicknesses of the cladding layer, the core diameter, the size of the elliptical cladding tube, and the diameter of the small circular cladding tube.

**Figure 1:**
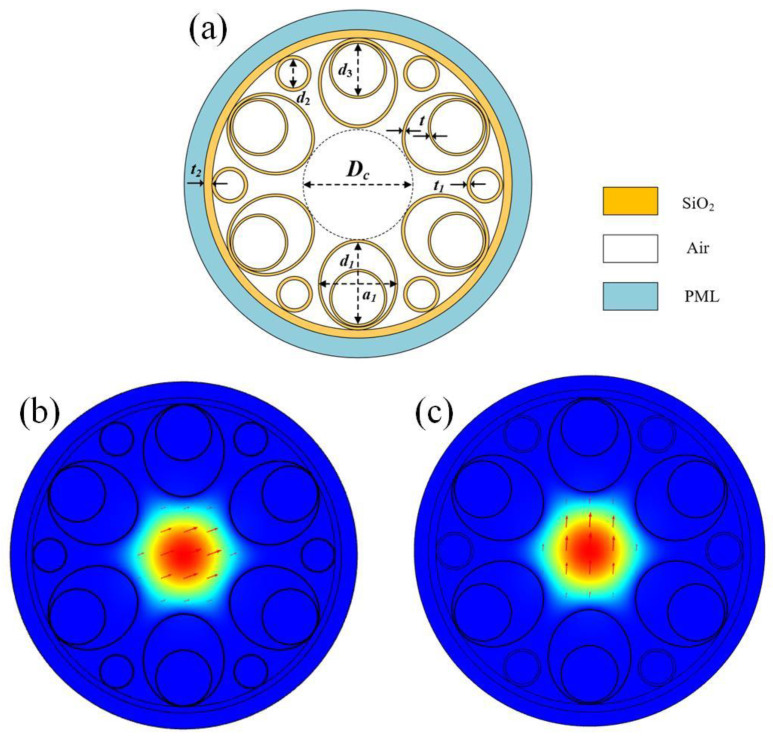
(**a**) Structural diagram of the designed HC-ARF; (**b**) X-polarized mode field distribution of the optical fiber at λ = 1550 nm; (**c**) Y-polarized mode field distribution of the optical fiber at λ = 1550 nm.

Figure 1a shows the cross-section of the HC-ARF structure with the number of cladding tubes N = 6, and each cladding tube contains one small circular cladding tube and one elliptical tube nested within a large circular cladding tube. The core diameter is *Dc*, the elliptical cladding tube has a long axis of *d*_1_ and a short axis of a_1_, the diameter of the large circular cladding tubes is *d*_3_, the diameter of the small circular cladding tubes is *d*_2_, the thickness of the elliptical and large circular cladding tubes is t, the thickness of the small circular cladding tubes is *t*_1_, and the thickness of the cladding layer is *t*_2_. The outermost layer is a perfectly matched layer (PML), typically set to be 2–3 times the wavelength. Both the cladding tubes and the cladding layer are silica, whose refractive index is wavelength-dependent and can be calculated using the Sellmeier equation [15].

According to the anti-resonance condition, when the wavelength satisfies the anti-resonance condition, the optical mode field is confined to the core region. However, when the wavelength satisfies the resonance condition, optical mode field leakage occurs. Figure 1b,c show the electric field distribution of the fundamental mode in the x-polarization and y-polarization, respectively, at the wavelength of 1550 nm. It can be observed that the optical mode field is well confined within the fiber core at the anti-resonance wavelength.

A full-vector finite element method combined with PML boundary conditions is adopted to analyze the properties of the proposed HC-ARF.

The confinement loss and the dispersion are the crucial parameters to measure the transmission characteristics of the HC-ARF, which can be calculated as [16,17]:

(1) $$CL=\frac{40\pi Im(n_{eff})}{\lambda In(10)}$$

(2) D=−λc×d2Re(neff)dλ2

where *n*_eff_ is the effective mode refractive index, *Im* (*n*_eff_) and *Re* (*n*_eff_) are the imaginary and real part of the effective mode refractive index, λ is the wavelength, and c is the speed of light.

The effective mode area is another important parameter to measure the performance of the fiber, which is [18]:

(3) $$A_{eff}=\frac{{\left(\iint_{\Omega }E^{2}dxdy\right)}^{2}}{\iint_{\Omega }E^{4}dxdy}$$

where *E* is the transverse component of the electric field propagating inside the fiber, and Ω is the cross section of the fiber.

## 3. Analysis of Results and Discussion

**Figure 2:**
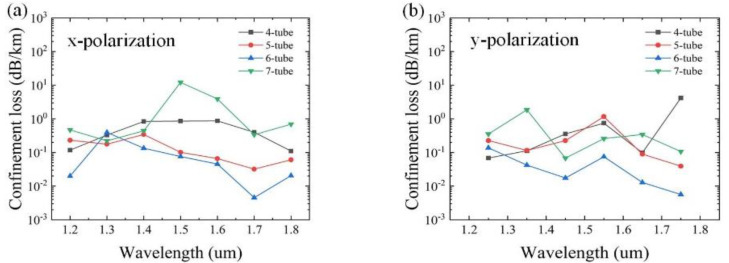
Variation of confinement loss for different numbers of tubes N. (**a**) x-polarization; (**b**) y-polarization.

The effects of the number of cladding tubes N, as the most important parameter, on the confinement loss are studied first. Based on the practical application requirements, the initial data are set as follows: *Dc* = 50 µm, *d*_1_= 40 μm, a_1_ = 35 μm, *d*_2_ = 14 μm, *d*_3_ = 24 μm, *t* = 0.5 μm, *t*_1_ = 1 μm, and *t*_2_ = 1.3 μm. The variation of the confinement loss as a function of the wavelength with different numbers of cladding tubes N = 4, 5, 6, and 7 for x and y polarizations is shown in Figure 2. It can be seen that the overall confinement loss is lowest for N = 6. This is mainly because the greater the number of cladding tubes, the stronger the ability to confine the light, which has lower confinement loss [19]. However, it is not the greater the number, the lower the confinement loss. The reason is that with the increase of N, the cladding tube gap will decrease, which can strengthen the resonance between the cladding tubes and cause increased confinement loss. Therefore, in the subsequent analysis, the number of cladding tubes N = 6 is adopted, as shown in Figure 1a.

**Figure 3:**
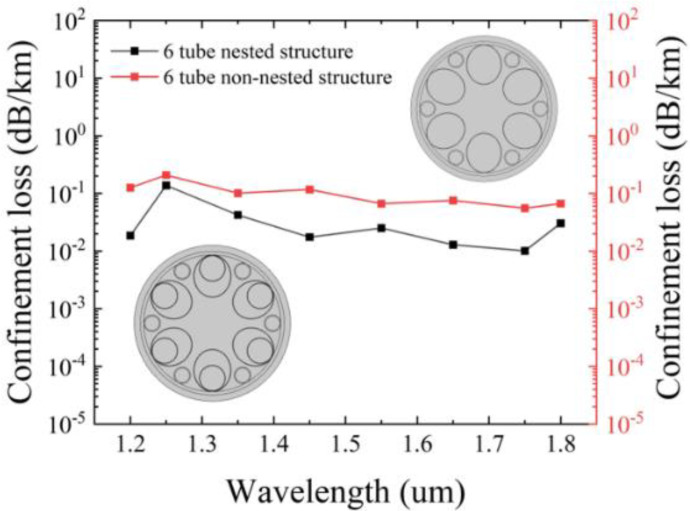
Variation of confinement loss in nested and non-nested fibers.

The reason why the nested cladding tubes are selected can be observed from Figure 3, which illustrates the variation of confinement loss with the nested and non-nested structures. It is evident that the nested tube structure exhibits lower confinement loss compared to the non-nested tube structure. It is easily understandable that adding nested tubes can effectively reduce the adverse effects caused by the gaps between the tubes, thereby increasing the ability to constrain light.

The influences of the parameters on the performance of the HC-ARF are investigated one by one within the wavelength range of 1200–2200 nm. The thickness of the elliptical and large circular cladding tubes, t, has the strongest effects, which can be calculated based on the following equation [20]:

(4) $$t=(2m+1)\lambda {\left[\begin{array}{c} 4{\left(n_{1}^{2}-n_{0}^{2}\right)}^{\frac{1}{2}} \end{array}\right]}^{-1}$$

where n_1_ represents the refractive index of silicon, *n*_0_ represents the refractive index of air, λ represents the wavelength, and m represents the order of resonance. The wavelength band is relatively wide with m = 1 and m = 2.

**Figure 4:**
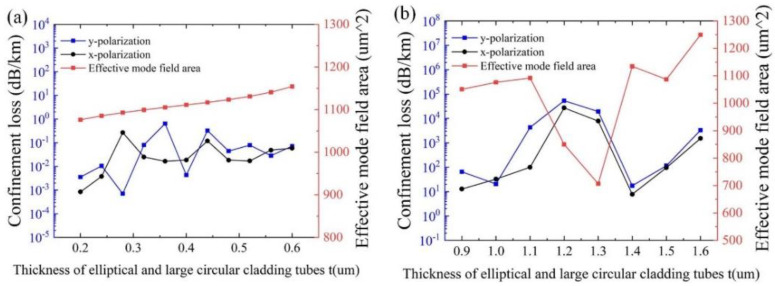
Variation of confinement loss and effective mode area with the thickness of the elliptical and large circular cladding tubes t at λ = 1550 nm. (**a**) when m = 1; (**b**) when m = 2.

Figure 4a,b show the effects of t on the confinement loss and the effective mode area with λ = 1550 nm for different m, and other initial parameters remain unchanged, respectively. As can be seen from Figure 4a, both the confinement losses and the effective mode area increase with the increase of t. The physical reason is that the narrowed gap between the cladding tubes, due to the increased t, can strengthen the resonance between the cladding tubes, resulting in increased confinement loss and effective mode area. It can be observed that the overall effective mode area remains above 1000 µm^2^, and the minimum confinement loss of 7.04 × 10^−4^ dB/km is obtained at t = 0.28 μm. As indicated in Figure 4b, the overall confinement losses deteriorate, with values above the order of 10. Thus, m = 1 is adopted.

**Figure 5:**
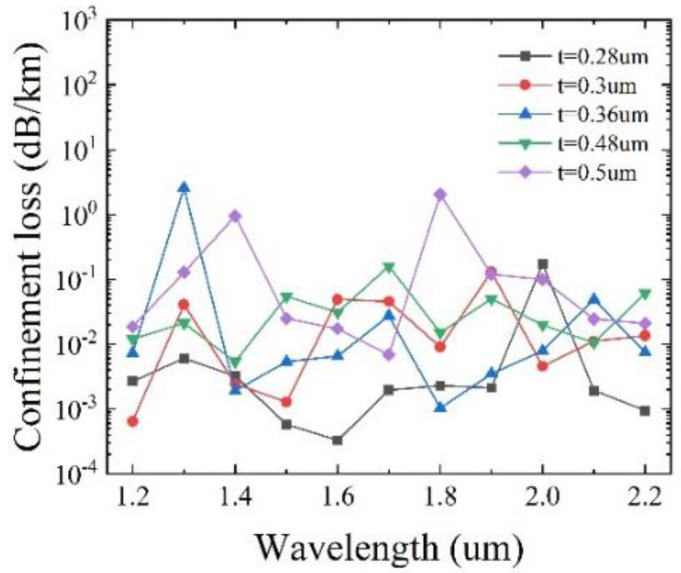
Variation of confinement loss with the wavelength for different thicknesses of the elliptical and large circular cladding tubes *t*.

To determine the optimal value of t, the confinement loss as a function of the wavelength for different t, with other initial parameters remaining unchanged, is shown in Figure 5. It can be seen that for *t* = 0.28 μm, the lowest loss of 2.79 × 10^−4^ dB/km is achieved at λ = 1450 nm, and the overall loss is below 0.39 dB/km. When *t* = 0.3 μm, the lowest loss of 6.45 × 10^−4^ dB/km is obtained at λ = 1200 nm with the overall loss below 0.13 dB/km. For *t* = 0.36 μm, the lowest loss of 9.43 × 10^−4^ dB/km is reached at λ = 1250 nm, with the overall loss below 3.09 dB/km. When *t* = 0.48 μm, the lowest loss of 3.8 × 10^−3^ dB/km is obtained at λ = 2150 nm, with the overall loss below 0.16 dB/km. For *t* = 0.5 μm, the lowest loss is 6.85 × 10^−3^ dB/km at λ = 1700 nm, with the overall loss below 2.04 dB/km. The comparison indicates that the lowest confinement loss can be achieved at *t* = 0.3 μm. As a consequence, the optimal t is determined to be 0.3 μm.

**Figure 6:**
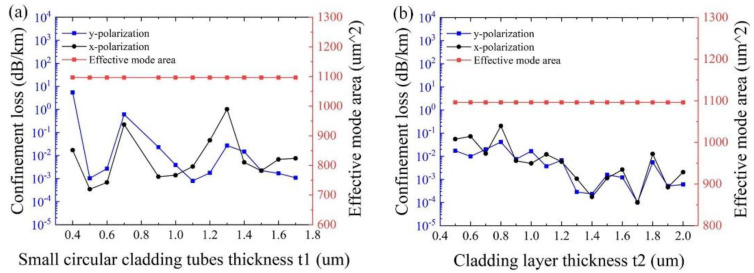
(**a**) Variation of confinement loss and effective mode area for different small circular cladding tubes thickness t_1_ at *λ* = 1550 nm. (**b**) Variation of confinement loss and effective mode area for different cladding layer thicknesses *t*_2_ at *λ* = 1550 nm.

Figure 6a,b show the influence of the thickness of small circular cladding tubes *t*_1_ and the cladding layer *t*_2_, with other initial parameters remaining unchanged, respectively. As can be seen from Figure 6a, the minimum confinement loss of 2.85 × 10^−4^ dB/km is obtained at *t*_1_ = 0.5 μm, and the effective mode area almost keeps stabilizing at ~1100 μm^2^. As shown in Figure 6b, the effects of the thickness of the cladding layer *t*_2_ on the effective mode area are negligible. The variation of the thickness of the cladding layer *t*_2_ has less impact on the confinement loss than that of the thickness of the elliptical and large circular cladding tubes t and the small circular cladding tubes *t*_1_. This is because the farther the distance from the core, the smaller the impact on the core mode. In particular, the minimum confinement loss of 1.03 × 10^−4^ dB/km is realized at *t*_2_ =1.7 μm for both polarizations. Therefore, it is reasonable to set *t*_1_ = 0.5 μm and *t*_2_ = 1.7 μm.

**Figure 7:**
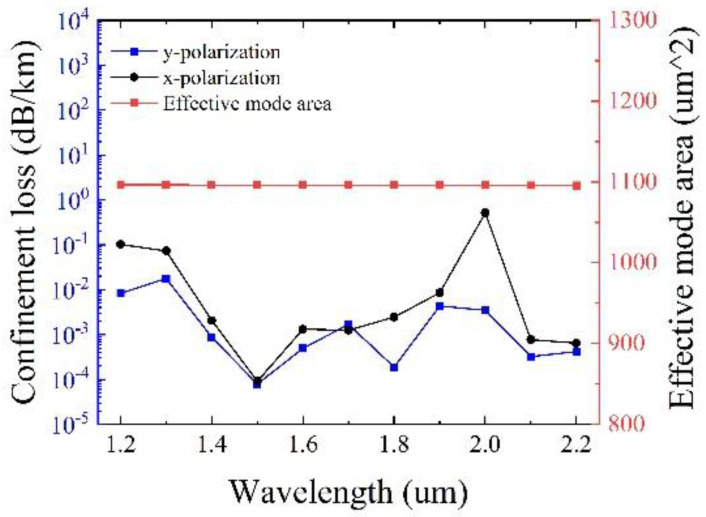
Variation of confinement loss and effective mode area with optimized tube thickness t, t_1_ and t_2_.

Figure 7 presents the confinement loss and the effective mode area as a function of the wavelength with optimized tube thickness *t*, *t*_1_ and *t*_2_ and other initial parameters remain unchanged. It can be observed that in the transmission band of 1000 nm, the confinement loss can reach the minimum value of 7.91 × 10^−5^ dB/km at 1500 nm, with a large effective mode area of 1096.3 μm^2^ simultaneously. It should be pointed out that the maximum confinement loss of y polarization is less than 0.018 dB/km, illustrating the excellent performance of low confinement loss.

**Figure 8:**
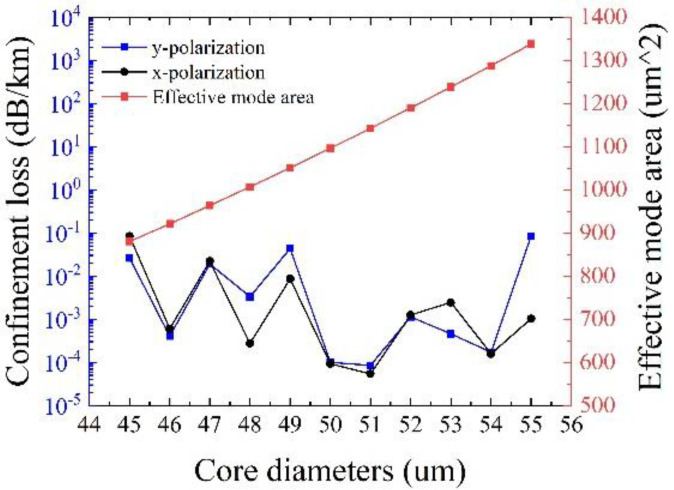
Variation of confinement loss and effective mode area with different core diameters.

Figure 8 shows the effects of the core diameter *Dc* on the confinement loss and the effective mode area at λ = 1550 nm, with other initial parameters remaining unchanged. It can be observed that the effective mode area increases with the core diameter, and the minimum confinement loss of 8.48 × 10^−5^ dB/km can be achieved at *Dc* = 51 μm, with a large effective mode area of 1142.5 μm^2^ together. It should be noted that it is not that the larger the core diameter, the lower the confinement loss. It has been proven that larger core diameters often pose challenges in ensuring single-mode transmission and may increase the losses of other higher-order modes in the fiber [21]. Consequently, *Dc* = 51 μm is adopted.

**Figure 9:**
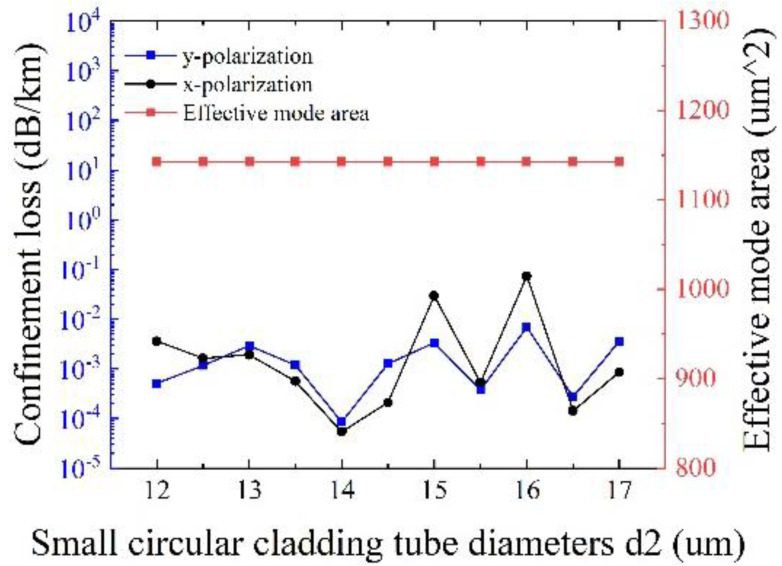
Variation of confinement loss and effective mode area corresponding to different small circular cladding tube diameters d_2_.

Figure 9 depicts the influences of the diameter of small circular cladding tubes *d*_2_ on the confinement loss and the effective mode area at λ = 1550 nm, with other initial parameters remaining unchanged. It is clear that the effects of *d*_2_ on the effective mode area can be neglected, and the maximum confinement loss is less than 0.1 dB/km. Especially, the minimum confinement loss of 5.42 × 10^−5^ dB/km for x-polarization and 8.48 × 10^−5^ dB/km for y-polarization can be realized at *d*_2_ = 14 μm with an effective mode area of 1142.5 μm^2^, demonstrating the realization of ultralow loss and large effective mode area at the same time. Therefore, the optimal value of *d*_2_ is 14 μm.

**Figure 10:**
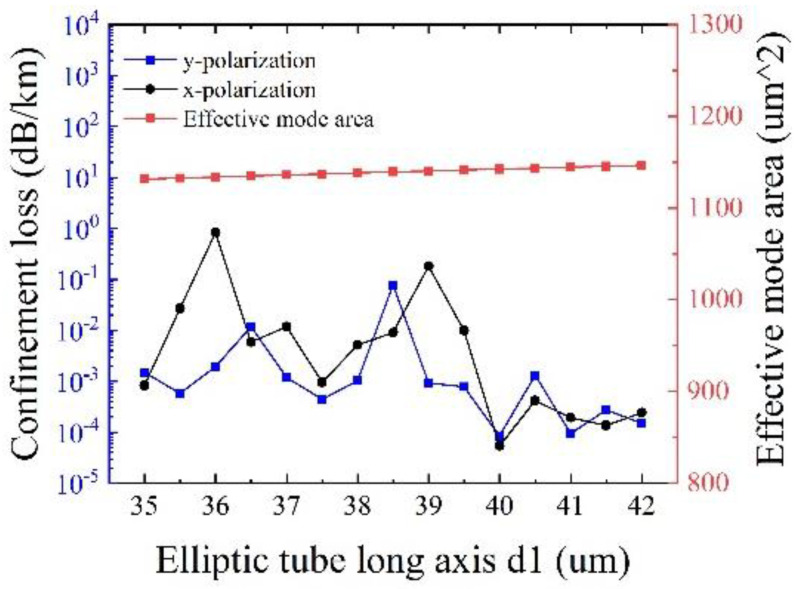
Variation of confinement loss and effective mode area corresponding to different small circular cladding tube diameters d_1_.

Figure 10 shows the effects of the long axis of the elliptical cladding tube *d*_1_ on the confinement loss and the effective mode area at λ = 1550 nm, with other initial parameters remaining unchanged. It can be seen that the effective mode area slightly increases with *d*_1_, and the minimum confinement loss of 5.42 × 10^−5^ dB/km is obtained at *d*_1_ = 40 μm for both polarizations, with an effective mode area of 1142.5 μm^2^. It is easier to understand that the increase of *d*_1_ can lead to easy mode coupling from the core to the cladding, thereby to increase the effective mode area. Hence, the optimized *d*_1_ is determined to be 40 μm.

**Figure 11:**
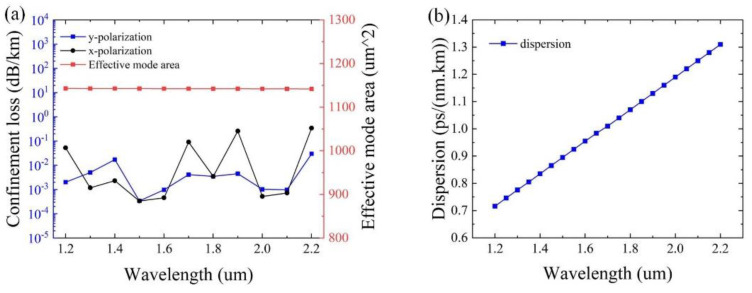
(**a**) Final optimization results of designed HC-ARF. (**b**) The dispersion of designed HC-ARF.

Based on the optimized parameters of *t* = 0.3 μm, *t*_1_ = 0.5 μm, *t*_2_ = 1.7 μm, *Dc* = 51 μm, *d*_2_ = 14 μm, and *d*_1_ = 40 μm, the optimization of the confinement loss and the effective mode area as a function of the wavelength is given in Figure 11. From Figure 11a, it can be observed that the effective mode area of ~1142 μm^2^ can be achieved, and the confinement loss is below 0.017 dB/km for y-polarization. Particularly, the minimum confinement loss of 1.2 × 10^−4^ dB/km is obtained at λ = 1500 nm for y-polarization. As can be seen from Figure 11b, the value of the dispersion ranges from 0.7 to 1.4 ps/(nm.km), exhibiting excellent dispersion characteristics of the designed HC-ARF, implying that the optimized structure has the ability to realize ultralow loss and a large effective mode area with excellent dispersion characteristics within a wide wavelength range simultaneously.

The preparation technology of optical fibers is a crucial aspect of development. It has been indicated that the HC-ARFs offer advantages such as a simple structure and feasible manufacturing, making them more suitable for practical use. The commonly used preparation processes for HC-ARFs include stacking, extrusion, and 3D printing, among which the bundle stacking method is particularly well-suited for the practical fabrication of the optical fiber designed in this study. The process involves stacking multiple preform optical fibers and subjecting them to thermal treatment. This technique uses thermal expansion and curing processes to form a hollow-core and anti-resonant structures. By controlling the arrangement, the stacking method, and the thermal treatment of the optical fibers, it is possible to achieve the desired parameters such as hollow-core diameter, periodicity, and anti-resonant structure dimensions under certain conditions. However, it has been found that errors may occur in HC-ARFs when using the stacking method, including deformation, collapse, and displacement of the anti-resonant tubes. Among these, the displacement of the anti-resonant tubes has the least impact on the optical fiber’s transmission performance, provided that an appropriate distance between the anti-resonant tubes is maintained during preparation. Thus, with the help of the stacking method and pressure-assisted joining technology, the new type of HC-ARF proposed in this paper can be successfully fabricated in the future, provided that the errors are kept minimal.

In order to illustrate the competitiveness of the proposed HC-ARF, the comparisons with other reported structures are listed in Table 1. It can be demonstrated that the HC-ARF presented in this paper exhibits better performance in terms of a wider transmission window, lower confinement loss, and dispersion value, as well as a larger effective mode area. In particular, the excellent characteristics of low confinement loss and dispersion, a large effective mode area, and a wide operating wavelength band can be realized simultaneously.

## 4. Conclusions

We have designed a novel hollow-core anti-resonant fiber that realizes a low confinement loss of less than 0.017 dB/km, a large effective mode area of ~1142 μm^2^, and a low dispersion of 0.7–1.4 ps/(nm·km) at the same time, in the operating wavelength range from 1200 nm to 2200 nm, with a wide transmission band of up to 1000 nm. The full-vector finite element method, combined with perfectly matched layer boundary conditions, has been adopted to analyze the influences of structural parameters on the performance of the fiber. It has been demonstrated that in the wavelength range of 1200–2200 nm, the confinement loss remains below 0.017 dB/km, and the minimum loss of 1.2 × 10^−4^ dB/km can be achieved at 1500 nm. At the commonly used communication wavelength of 1550 nm, the confinement loss is as low as 1.73 × 10^−3^ dB/km. The simultaneous achievement of low loss and a large effective mode area, along with excellent dispersion characteristics in a wide wavelength band, illustrates the outstanding performance of the proposed HC-ARF. The new design has the ability to provide valuable insights for the practical application of low-loss and large mode area hollow-core anti-resonant fibers to meet the requirements of long-distance, high-power transmission with a large transmission window simultaneously.

## Figures and Tables

**Table 1:** Comparison of the performance of proposed HC-ARF with other reported HC-ARFs.

Ref.	Fiber Structure	Performance Metrics					
		Transmission Window (μm)	Overall Confinement Loss (dB/km)	Minimum Confinement Loss (dB/km)	Dispersion (ps/(nm·km))	Effective Mode Area (μm^2^)	Reference
2019	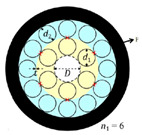	(200) 1000–1200	<0.1	~	~	~	[22]
2022	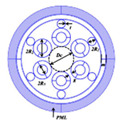	(640) 1140–1780	<0.005	4 × 10^−4^@1550	~	~	[21]
2022	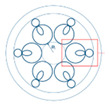	(365) 1260–1625	<0.01	4 × 10^−4^@1550	~	~	[14]
2023	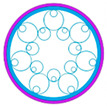	(100) 1525–1625	<10	0.016@1550	−5	~	
2023	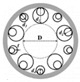	(500) 1200–1700	≤0.065	0.039@1500	1.020–1.222	750	
This work	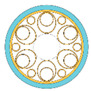	(1000) 1200–2200	<0.017	1.2 × 10^−4^@1500	0.7–1.4	1145	

## Data Availability

Data and code of this work will be available from the corresponding author upon reasonable request.
